# Burden of acne vulgaris, psoriasis, atopic dermatitis, and urticaria in individuals under 20 years of age in China and globally, 1990-2021: A systematic analysis with 10-year forecast

**DOI:** 10.1016/j.jdin.2025.11.018

**Published:** 2025-11-29

**Authors:** Jiawen Wu, Yuanqin Wang, Yuxin Qing, Bingyang Xu, Yan Yang, Hong Sun, Na Wu

**Affiliations:** a Department of Dermatology, The Second Affiliated Hospital of Xi'an Jiaotong University, Xi'an, China; b Department of Clinical Medicine, The Second School of Clinical Medicine, Xi’an Jiaotong University Health Science Center, Xi’an, Shaanxi, China; c Department of Neurology, The Second Affiliated Hospital of Xi'an Jiaotong University, Xi'an, China; d Department of Nursing, Xian Jiaotong University Medical School, Xi'an, China

**Keywords:** acne vulgaris, atopic dermatitis, burden of disease, joinpoint regression analysis, psoriasis, urticaria

*To the Editor:* Acne vulgaris, psoriasis, atopic dermatitis (AD), and urticaria are the most common nonfatal diseases in youth, profoundly impacting quality of life.^1^ While a recent Global Burden of Disease study^2^ quantified the current prevalence, long-term projections of the global and China-specific trends among individuals aged <20 years remain scarce. In this study, we comprehensively analyzed the burden of these 4 diseases among such individuals by using the 2021 Global Burden of Disease data, with projections extending to 2031.^3^

We discovered that the disease burdens of psoriasis, AD, and urticaria among Chinese adolescents were lower than global averages. Acne vulgaris and psoriasis peaked in adolescents aged 15-19 years, AD in those aged 5-9 years, and urticaria in children aged <5 years. The age-standardized prevalence rates (ASPRs) of acne vulgaris in the 15-19-year age group globally and in China were 2714.68 (95% uncertainty interval: 2318.75-3144.82) and 2831.30 (95% uncertainty interval: 2351.11-3349.066) per 100,000 people, respectively, and the estimated annual percentage change in age-standardized years lived with disability rates were 12.87% and 15.27%, respectively (Table I). The disease burdens of all 4 conditions in females exceeded those in male patients. A higher sociodemographic index positively correlated with the psoriasis and AD burden; conversely, acne vulgaris and urticaria were disproportionately concentrated in low-sociodemographic index regions (Supplementary Materials, available via Mendeley at https://data.mendeley.com/preview/f5z42fssm6?a=986ce001-d75a-4186-89b8-0c80c7f21ce2).

**Table I:** The number and age-standardized rates of prevalence and YLDs on acne vulgaris among children and adolescents under 20 years globally and in China in 1990 and in 2021 and its EAPC from 1990 to 2021

Disease	Measure	Sub - measure	China		EAPC	Global		EAPC
			ASR (95% UI) in 1990	ASR (95% UI) in 2021		ASR (95% UI) in 1990	ASR (95% UI) in 2021	
Acne vulgaris	Prevalence		6043.5 (6041.29-6045.71)	7133.71 (7130.88-7136.54)	15.28%	5133.2 (5132.27-5134.14)	5926.7 (5925.79-5927.61)	13.39%
		Sex						
Acne vulgaris	Prevalence	Female	7577.29 (7573.73-7580.86)	9024.0072 (9019.35-9028.67)	16.03%	5782.85 (5781.43-5784.27)	6603.78 (6602.4-6605.16)	12.43%
Acne vulgaris	Prevalence	Male	4601.62 (4598.95-4604.3)	5488.48 (5485.087-5491.88)	16.16%	4509.87 (4508.64-4511.098)	5288.17 (5286.97-5289.37)	14.72%
		Age						
Acne vulgaris	Prevalence	<5	0	0	0	0	0	0
Acne vulgaris	Prevalence	5-9	381.39 (309.65-448.41)	457.32 (370.15-532.39)	16.60%	166.58 (136.81-197.068)	185.75 (152.35-218.23)	10.32%
Acne vulgaris	Prevalence	10-14	2199.3 (1854.23-2536.41)	2589.26 (2181.23-2980.85)	15.06%	1697.56 (1451.95-1966.76)	1982.92 (1697.92-2293.47)	14.39%
Acne vulgaris	Prevalence	15-19	2398.9 (1987.35-2872.06)	2831.3 (2351.11-3349.066)	15.27%	2365.41 (2005.32-2767.89)	2714.68 (2318.75-3144.82)	12.87%
Acne vulgaris	YLDs		130.72 (130.39-131.045)	154.57 (154.15-154.99)	15.43%	110.28 (110.14-110.42)	127.41 (127.27-127.54)	13.44%
		Sex						
Acne vulgaris	YLDs	Female	163.78 (163.25-164.3)	195.43 (194.75-196.12)	16.20%	124 (123.79-124.21)	141.59 (141.39-141.79)	12.42%
Acne vulgaris	YLDs	Male	99.65 (99.25-100.041)	119.00039 (118.5-119.5)	16.26%	97.12 (96.94-97.3)	114.034 (113.86-114.21)	14.83%
		Age						
Acne vulgaris	YLDs	<5	0	0	0	0	0	0
Acne vulgaris	YLDs	5-9	8.29 (5.0067-13.35)	9.97 (6.12-16.097)	16.78%	3.61 (2.15-5.77)	4.037 (2.46-6.44)	10.46%
Acne vulgaris	YLDs	10-14	47.6 (29.42-74.48)	56.14 (34.56-88.041)	15.22%	36.54 (22.25-56.83)	42.71 (26.1-66.35)	14.46%
Acne vulgaris	YLDs	15-19	51.82 (31.45-83.51)	61.25 (37.72-99.62)	15.40%	50.72 (31.028-81.9)	58.23 (35.8-94.29)	12.90%

*ASR*, Age-standardized rate; *EAPC*, estimated annual percentage change; *UI*, uncertainty interval; *YLD*, years lived with disability.

Our projections indicate that, by 2031, the global ASPRs of acne vulgaris, psoriasis, and urticaria will rise to 8730.54, 224.76, and 1216.18 per 100,000 people, respectively, and those in China to 9791.88, 215.16, and 1001.78 per 100,000 people, respectively. However, the global ASPRs of AD are projected to decrease to 3158.32 per 100,000 people, while those in China are projected to rise to 2806.30 per 100,000 people. Thus, the age-specific patterns may persist through 2031 (Fig 1).

**Fig 1:**
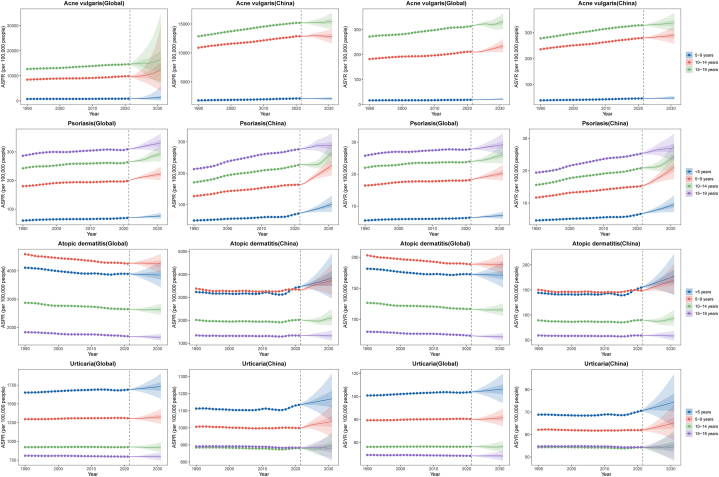
Prediction of the age trends to age-standardized rate for skin diseases in children and adolescents populations among age group under 20 globally and in China until to 2031.

Skin disease epidemiology now encompasses not only biology but also environmental exposure, behavioral patterns, and health care capacity. The rise in the acne vulgaris burden is evident in China. Similar patterns have been reported in other rapidly urbanizing countries in Asia and Latin America. This suggests that the interplay between socioeconomic transition, digital immersion, and metabolic stress may represent a broader phenomenon affecting adolescent skin health worldwide.^1^ In contrast, the decreasing burden of AD and urticaria with age suggests a stronger influence of early-life environmental exposures.^4^ However, natural immune maturation and improved self-care behaviors with age may contribute to symptom remission over time. We discovered that Chinese adolescents had a significantly higher rate of psoriasis growth than the global adolescent population. The observed increase in particularly the earlier recognition of comorbidities (metabolic syndrome and mental disorders) in youth likely reflects improved diagnostic awareness rather than solely an increase in the disease incidence.^5^

Long-term projections carry uncertainties owing to model assumptions and data variability; notably, our models did not account for black swan events (eg, pandemics) that may abruptly reshape disease landscapes. In response to the trends shown in this study, policymakers should prioritize early prevention while adapting to dynamic societal changes.

## Conflicts of interest

None disclosed.
